# Propensity score matching evaluation of psychological stress and hair cortisol among people living with HIV in China

**DOI:** 10.1038/s41598-021-90922-z

**Published:** 2021-06-01

**Authors:** Xu Chen, Shuaifeng Liu, Chengbo Zeng, Xiaoming Li, Shan Qiao, Riying Lv, Zhiyong Shen

**Affiliations:** 1 Department of Respiratory and Critical Care Medicine, Nanxishan Hospital of Guangxi Zhuang Autonomous Region, Guilin, Guangxi China; 2 Guangxi Center for Disease Prevention and Control, Nanning, Guangxi China; 3 Department of Health Promotion Education and Behavior, School of Public Health, University of South Carolina, Columbia, SC USA; 4 Department of Infectious Diseases, Guigang City People’s Hospital, Guigang, Guangxi China

**Keywords:** Psychology, Diseases, Endocrinology

## Abstract

To compare the psychological stress level and hair cortisol level of people living with HIV (PLWH) with those without HIV in China, a total of 220 participants were initially enrolled in the study, including 200 PLWH and 20 people living without HIV. Psychological stress level, including quality of life, anxiety, perceived stress and psychological resilience, was self-reported in both groups with related scales. The cortisol in hair was extracted and assessed by LC-APCI-MS/MS method. Propensity score matching analysis was performed to balance the baseline covariates of the two groups, whereas the difference in psychological stress level and hair cortisol level between the two groups was compared. Furthermore, the associations between psychological stress level and cortisol level were examined. Two comparison groups were matched by 1:3 propensity score matching, which yielding 20 people living without HIV and 60 PLWH. Ultimately, in regarding to the psychological stress, the levels of the anxiety (34 vs. 26, *p* < 0.001), perceived stress (38.5 vs. 33, *p* = 0.001) and psychological resilience (31 vs. 26, *p* = 0.004) were higher among PLWH than those living without HIV, but the people without HIV showed higher quality of life (109 vs.116, *p* < 0. 001). The hair cortisol level (34.66 vs. 21.61, *p* = 0.002) in PLWH was higher than those living without HIV. However, there were no significant associations between psychological stress level and cortisol level (*p* > 0.05). The PLWH showed higher level of psychological stress and cortisol than those without HIV. No relationship was seen between psychological stress level and cortisol level in PLWH.

## Introduction

Human immunodeficiency virus (HIV), which causes Acquired Immunodeficiency Syndrome (AIDS), affects up to 0.8% of the adult worldwide. Over the past decades, international donor institutions and governments have allocated substantial resources to control the HIV/AIDS epidemic. Despite the significant progress in this process, the epidemic still represents a significant public health threat^1^, with an estimated 37.9 million of PLWH by the end of 2018 worldwide^2^. The number of HIV-infected patients is still rising in China and other parts of the world. With the antiretroviral therapy (ART), there was a significant decline in the incidence of opportunistic infection- and neoplasm- related diseases. Nevertheless, psychological stress, including anxiety, perceived stress and stigma, as well as HIV-related endocrinopathies seem to become increasingly important among PLWH^3^. Previous studies have shown that nearly 50% of the PLWH suffer from discrimination, depression, anxiety, perceived stress and other psychological stresses at all stages of HIV infection^4^. The high levels of psychological stress would generate physiological reactions (e.g. cortisol, dehydroepiandrosterone, catecholamine) and harm the physical health of PLWH.

Cortisol, as one end product of the hypothalamic–pituitary–adrenal (HPA) axis, has been widely used as one of the main indicators of cumulative physiological responses associated with psychological distress^5,6^. These responses are generated when people suffer from emergent external stress and help them adapt to the special environmental changes. Cortisol could be determined from the blood, saliva, and urine samples. Because these common sampling matrices provide measures of cortisol concentration at single point in time, they are likely affected by the time of samples collected, individual differences in circadian rhythms, and transient exposure to acute daily stressors. Compared with those traditional measurements, hair cortisol is relatively stable and could capture the long-term physiological responses related to stress. What’s more, hair samples can be stored for long periods and delivered without biohazardous precautions or cold chain requirements^7^. Therefore, hair cortisol measurement is likely to overcome the shortcomings mentioned above. It is not surprising that hair cortisol is a potentially promising biomarker of psychological distress.

Exploring the differences in psychological stress and hair cortisol between healthy people and PLWH and the relationship between psychological stress and hair cortisol could greatly facilitate for screening sensitive indices that are potentially used as important psychological reference of clinical diagnosis. Therefore, we conducted this study to investigate the psychological stress level and cortisol level among PLWH from China. In contrast to previous studies using single time point (saliva, serum) or short-term (24 h urine) cortisol measurements, we used a relatively novel but well-validated method to determine long-term systemic cortisol levels in scalp hair^8^. Importantly, psychological stress and hair cortisol levels are likely to be affected by various confounders, such as age, gender, body mass index (BMI), and ethnicity, which can bias the results. We therefore performed propensity score matching (PSM) analysis to balance baseline covariates between the groups of PLWH and people living without HIV to examine the relationship between HIV infection and psychological distress, as well as level of cortisol. The associations between psychological distress and cortisol were examined as well.

## Results

### Baseline characteristics

**Figure 1:**
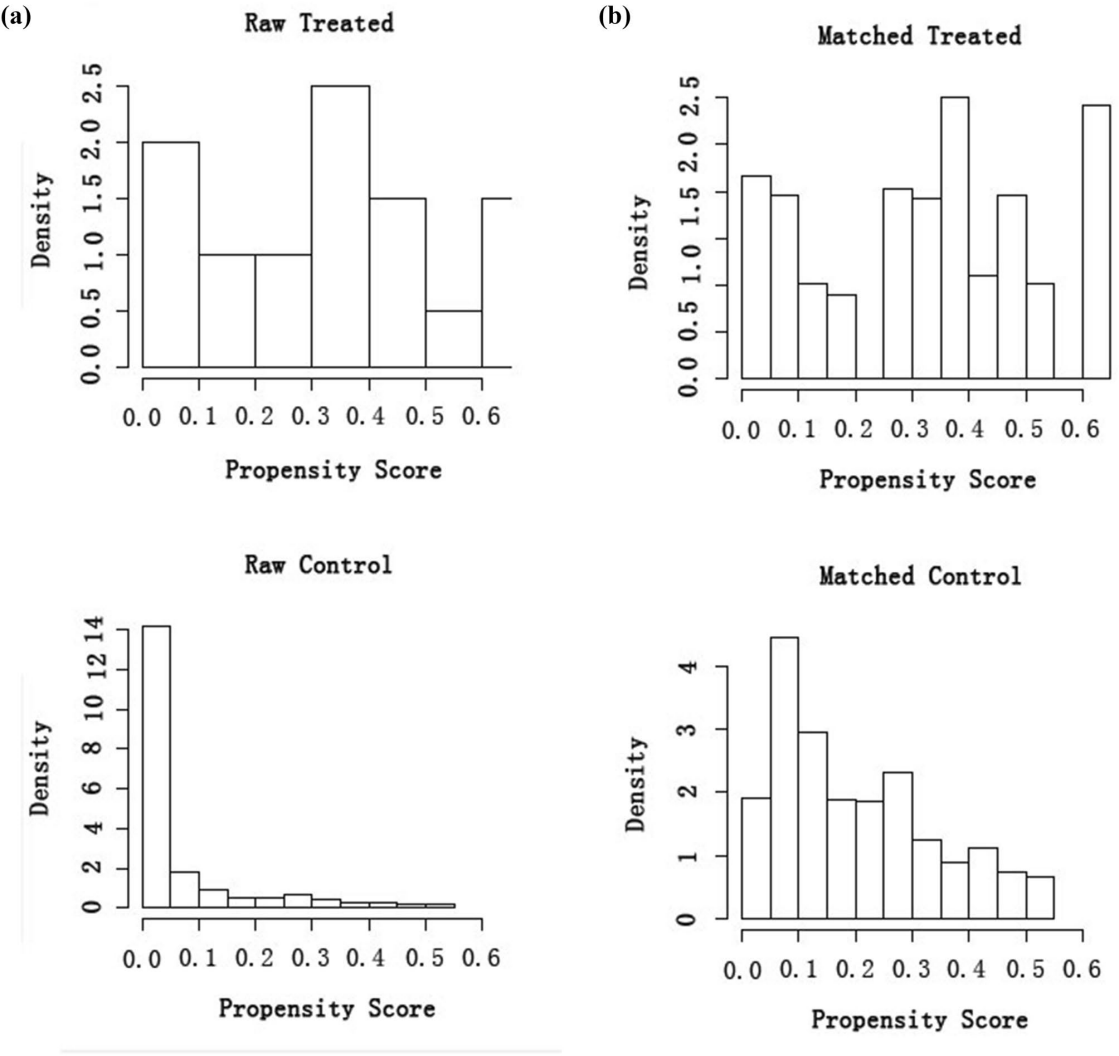
Histograms of the propensity score distribution. (**a**) Histograms of the unmatched participants. (**b**) Histograms of the matched participants. Note: The histograms were created via R package “Matching”. Note: The overall balance of the thirteen variables in the model indicates that the matching process was successful**.**

A total of 220 participants were enrolled in the study, including 20 people living without HIV and 200 PLWH. Among them, 137 (62.27%) of the participants were males while 83 (37.73%) were females. The median age was 41.73 (IRQ: 33.94–48.68) years old and the median BMI was 21.30 (IRQ: 19.77–23.82). Before matching, the baseline characteristics between the two groups that differed significantly in terms of ethnicity, monthly household income and alcohol use (*p* < 0.05). We used the propensity score matching method to balance the differences between the study and control groups. The 1:3 PSM yielded matched pairs of 20 participants in the control group and 60 PLWH in the study group. No statistical differences were observed between the two groups with *p* > 0.05 for all baseline variables after matching (Table 1). In order to further study this imbalance, histograms were used to display the propensity score distribution before and after PSM. Figure 1a presents a histogram showing an unbalanced distribution of propensity scores for the overall participants, whereas Fig. 1b presents the tendency for equilibrium of the score distribution in the histogram for the matched participants.

**Table 1:** Baseline characteristics before and after propensity matching in control and study groups.

Characteristics	Before propensity matching			After propensity matching		
	Control group N = 20	Study group N = 200	*P* value	Control group N = 20	Study group N = 60	*P* value
**Age (median, IQR)**	39 (32.8–44.3)	42 (34.1–49.1)	0.088	39 (32.8–44.3)	37 (31–47.3)	0.534
**Gender**			0.482			1.000
Male	11 (55%)	126 (63%)		11 (55%)	33 (55%)	
Female	9 (45%)	74 (37%)		9 (45%)	27 (45%)	
**BMI (median, IQR)**	21.3 (19.7–22.6)	21.3 (20.0–24.0)	0.857	21.3 (19.7–22.6)	21.2 (19.8–22.6)	0.874
**Ethnicity**			0.030			1.000
Han	19 (95%)	146 (73%)		19 (95%)	55 (91.7%)	
Non-Han	1 (5%)	54 (27%)		1 (5%)	5 (8.3%)	
**Marital status**			0.427			1.000
Married	16 (80%)	173 (86.5%)		16 (80%)	50 (83.3%)	
Never married	4 (20%)	27 (13.5%)		4 (20%)	10 (16.7%)	
**Education level**			0.614			0.700
Illiterate or primary school	5 (25%)	70 (35%)		5 (25%)	17 (28.3%)	
Junior high school	10 (50%)	85 (42.5%)		9 (45%)	28 (46.7%)	
Senior high school	3 (15%)	34 (17%)		4 (20%)	13 (21.7%)	
College or above	2 (10%)	11 (5.5%)		2 (10%)	2 (3.3%)	
**Employment status**			0.082			NA
Unemployed	0	18 (9%)		0	0	
Part time employed	0	24 (12%)		0	0	
Full time employed	20 (100%)	158 (79%)		20 (100%)	60 (100%)	
**Monthly household income (Yuan)**			0.001			0.270
< 2000	0	32 (16%)		0	0	
2000–2999	4 (20%)	95 (47.5%)		4 (20%)	22 (36.7%)	
≥ 3000	16 (80%)	73 (36.5%)		16 (80%)	38 (63.3%)	
**Tobacco use**			0.064			0.636
Yes	3 (15%)	71 (35.5%)		3 (15%)	14 (23.3%)	
No	17 (85%)	129 (64.5%)		17 (85%)	46 (76.7%)	
**Alcohol use**			0.012			0.819
Yes	1 (5%)	64 (32%)		1 (5%)	6 (10%)	
No	19 (95%)	136 (68%)		19 (95%)	54 (90%)	
**Frequency of hair washing**			0.746			1.000
Once every 1 to 3 days	18 (90%)	175 (87.5%)		18 (90%)	52 (86.7%)	
Once every 4 to 7 days	2 (10%)	25 (12.5%)		2 (10%)	8 (13.3%)	
**Using Hair dryer, curling iron or hair straightener**			0.895			0.896
Yes	8 (40%)	77 (38.5%)		8 (40%)	27 (45%)	
No	12 (60%)	123 (61.5%)		12 (60%)	33 (55%)	
**Using hair styling products**			0.261			NA
Yes	0	12 (6%)		0	0	
No	20 (100%)	188 (94%)		20 (100%)	60 (100%)	

Data are presented as n(%) or median (25th–75th interquartile range).

### Comparison of psychological stress and hair cortisol between control and study groups

**Figure 2:**
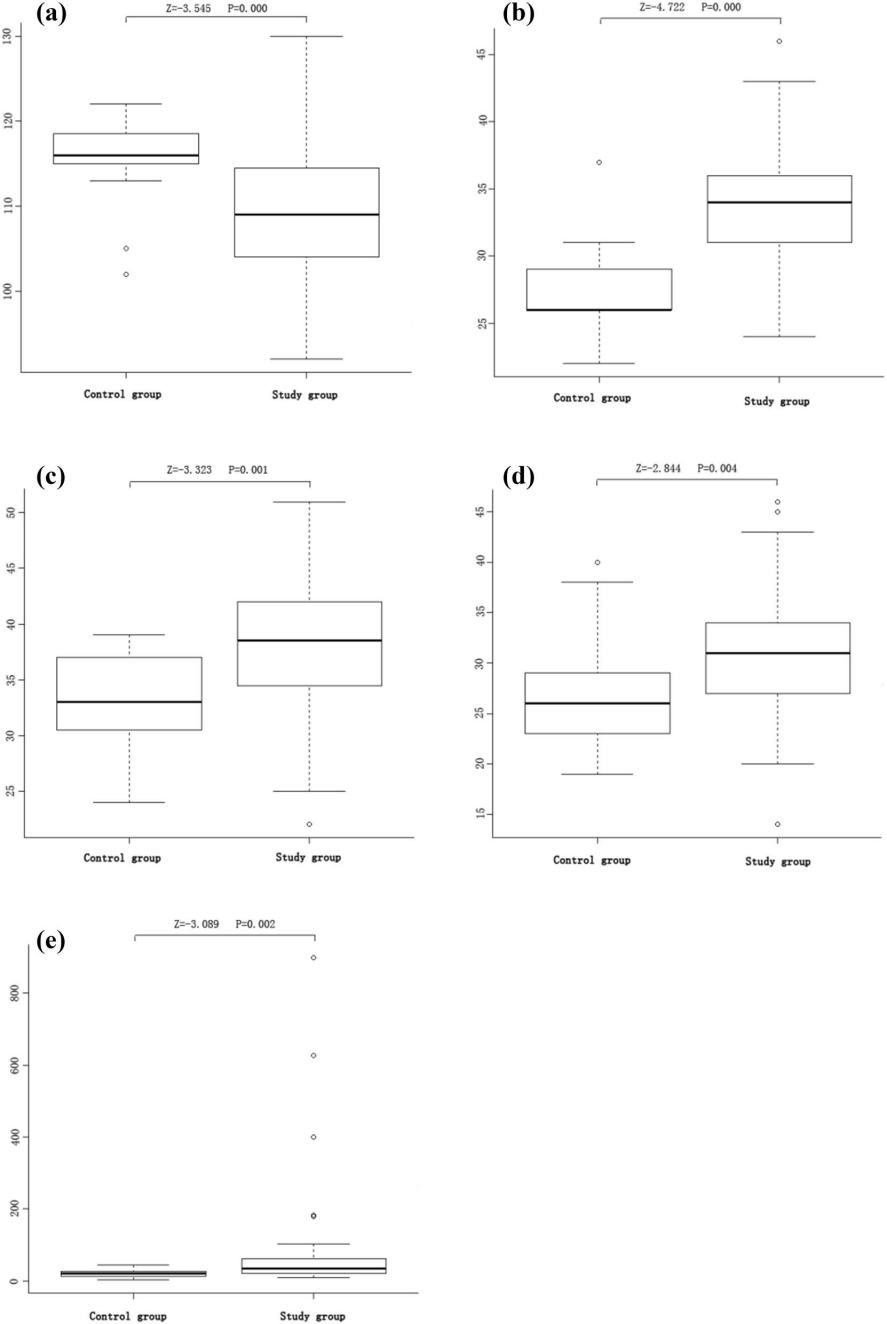
Comparison of psychological stress level and cortisol level between control and study groups after propensity matching. (**a**) The level of quality life among PLWH and people without HIV, (**b**) The level of anxiety among PLWH and people without HIV. (**c**) The level of perceived stress among PLWH and people without HIV. (**d**)The level of psychological resilience among PLWH and people without HIV. (**e**) The level of cortisol among PLWH and people without HIV. Note: The box-plots were created via R package “Vegan”.

In this study, we compared the levels of psychological stress and hair cortisol between the study and control groups (Table 2). The medians of psychological stress are presented in Fig. 2a–d. Compared with control group, the study group reported significantly lower level of quality of life (109 vs.116, *p* < 0.001). In addition, the study group reported significantly higher levels of anxiety (34 vs. 26, *p* < 0.001), perceived stress (38.5 vs. 33, *p* = 0.001) and psychological resilience (31 vs. 26, *p* = 0.004) than control group. In PLWH, their median of hair cortisol was 34.66 (IQR: 21.74–61.93), whereas for the participants in the control group, their median of hair cortisol was 21.61 (IQR: 14.83–26.75), and the difference in hair cortisol was statistically significant between the two groups (*p* = 0.002) (Fig. 2e).

**Table 2:** Comparison of psychological stress and hair cortisol between control and study groups.

	Control group	Study group	Z value	*P* value
**Psychological measure**				
Quality of life	116 (115–118.25)	109 (104–114.25)	− 3.545	0.000
Anxiety	26 (25.75–29)	34 (31–36)	− 4.722	0.000
Perceived stress	33 (30.75–37)	38.5 (34.75–42)	− 3.323	0.001
Psychological resilience	26 (23–28.5)	31 (27–34)	− 2.844	0.004
**Endocrine measure**				
Cortisol	21.61 (14.83–26.75)	34.66 (21.74–61.93)	− 3.089	0.002

### The associations between psychological stress and cortisol

According to Spearman correlation analyses, hair cortisol was negatively related to quality of life (r = − 0.063, *p* = 0.378) and perceived stress (r = − 0.043, *p* = 0.542). However, it was positively related to anxiety (r = 0.061, *p* = 0.389) and psychological resilience (r = 0.082, *p* = 0.249). But none of these associations were statistically significant.

Medians for each psychological stress by hair cortisol level are illustrated in Table 3. Hair cortisol level was negatively associated to quality of life (z = − 0.005, *p* = 0.996), anxiety (z = − 0.724, *p* = 0.469), perceived stress (z = − 5.04, *p* = 0.614) and psychological resilience (z = − 1.139, *p* = 0.255) although no significant association was detected.

**Table 3:** Psychological stress by level of hair cortisol.

Cortisol	Quality of life	Anxiety	Perceived stress	Psychological resilience
High (≤ 29.56)	109.5 (105–115.25)	34 (30–36)	38.5 (35–42)	31 (28–34)
Low (> 29.56)	110 (105–115)	33 (30–36)	38 (32–41)	29.5 (27–34.75)
Z value	− 0.005	− 0.724	− 5.04	− 1.139
*P* value	0.996	0.469	0.614	0.255

## Discussion

This study compared the psychological distress and hair cortisol between PLWH and people living without HIV from Guangxi, China, and showed that PLWH displayed higher levels of psychological stress and hair cortisol. Moreover, we found that the PLWH presented lower quality of life but higher psychological resilience. These results are consistent with that of the existing literature^9,10^. As we know, psychological distress and hair cortisol are likely to be affected by various confounders. The propensity score matching analysis was used in our study to minimize the confounding factors as far as possible. A previous study has suggested that matching according to the propensity score eliminates a greater proportion of baseline difference between any two groups compared to stratification or covariate adjustment^11^. The 1-to-3 propensity score matching provides a robust evaluation of psychological stress and hair cortisol among PLWH.

The results in this study revealed that PLWH showed higher level of psychological stress compared with the general population, which are the same as seen in previous study^12^. A study from the United States showed that about 56–78% of AIDS patients suffer from mental disorders. Depression, anxiety, fear, loss of confidence in life, suicidal behaviors are common mental health disorders among them^13^. A large number of researches indicated that the PLWH presented significant lower level of psychological health than those without HIV^14,15^. Most PLWH must cope with the complex and multiple psychological problems (e.g. depression, perceived stress, stressful life events) of a life-threatening illness and its treatment, which simultaneously facing financial hardship, life-style associated social stigmas, multiple bereavements, and other personal losses^16^.With the high prevalence of these stressors, it is not surprising that PLWH are at increased risk for affective disorders and sustained high levels of psychological stress.

Our study also found that the PLWH displayed higher level of hair cortisol than those without HIV. The reason is, on the one hand, that HIV can activate monocytes-macrophages to produce cytokines including interleukin-1, tumor necrosis factor, interferon and then directly or indirectly influence the HPA to stimulate the secretion of cortisol^10,17^. On the other hand, cortisol is an important indicator of chronic stress. As mentioned above, PLWH have high levels of psychological distress, which promote the secretion of adrenocorticotrophic hormone resulting in the increased cortisol level^18^. In contrast, some studies indicated that 8–14% PLWH were observed lower level of cortisol than normal level^17,19^. It was likely to relate to activation of polyclonal β cell, development of anti-adrenal cell antibody and adrenal dysfunction^20^. Other factors including diet, sports and drugs, which were difficult to be excluded, may also affect the synthesis of cortisol as well.

Cortisol is commonly known as the stress hormone because it is released in higher doses under stressful condition. For instance, some studies indicated that hair cortisol increased with higher level of stressful life events, depression, and posttraumatic stress disorder^21–23^. In the same way, one systematic literature review on hair cortisol and stress exposure indicated that effect sizes for the connections between hair cortisol and chronic stress were mostly medium and large^24^. However, although PLWH had higher level of psychological stress and cortisol than the control group, no significant association between psychological stress and hair cortisol was found in the study, which was consistent with previous studies^25,26^. Some studies even reported that anxiety was negatively associated with hair cortisol^27^. Several factors should be considered in interpreting our result. The HPA axis is a highly reactive system which is easily affected by a wide array of factors. Previous evidence suggests that solely the situation of taking part in scientific research may present a factor that can acutely affect adrenocortical activity, e.g. by leading to changes in normal daily routines^28^. It is conceivable that the extent of such acute influences on cortisol levels might not be uniform across participants. What’s more, some participants were receiving concomitant psychotropic medications and / or antiviral agent, which also presenting confounding influence on the result.

Several limitations should be recognized in this study. First, the small sample size in this study might limit the statistical power of the analysis. Second, the cortisol and psychological situation were affected by various confounders. Although we used PSM to balance some of the sociodemographic characteristics between study and control groups, we could not completely exclude the influence of all confounders. Third, the study may have suffered from non-respondents bias as there were some participants who refused to participate in the study^29^. Nevertheless, the study enabled us to understand the psychological distress and hair cortisol between the PLWH and the people without HIV in China. These findings may have implications for mental intervention and HIV-related endocrinopathies targeted at HIV-infected population. Special attention focused on psychological distress and cortisol should be given to PLWH. Though no relationship between psychological stress and cortisol was found in this study, hair cortisol analysis still hold great promise to accurately assess long-term levels of psychological distress, which could greatly contribute to early diagnosis and intervention of psychological health diseases and the improvement of mental wellbeing among PLWH. Further research is needed to better understand psychological illness and endocrine issues among the PLWH. Further studies investigating the relationship between the psychological stress and the cortisol are warranted as well.

In summary, our study confirms that the PLWH from China suffer from both higher levels of psychological stress and cortisol. Moreover, we find that the PLWH presented lower quality of life but higher psychological resilience. Further, no significant association was found between psychological stress and hair cortisol among PLWH in this study. The psychological distress and HIV-related endocrine system disease among PLWH are deserved more attention.

## Materials and methods

The study protocol was approved by the Guangxi Institutional Review Board. Written informed consent was obtained from participants. All of the procedures were performed in accordance with the Declaration of Helsinki and relevant policies in China.

### Subjects

All participants of this study were recruited from Department of Infectious Disease in People’s Hospital of Guigang City. Participants who were at least 18 but younger than 60 years of age were eligible for recruitment. The exclusion criteria were individuals who: (1) had confirmed symptoms of opportunistic infections; (2) endocrine diseases (e.g. macroamylasemia, adrenal dysfunction, hyperamylasemia, autoimmune thyroid diseases, etc.); (3) currently take hormonal drugs (e.g. prednisolone) which might influence the cortisol level; (4) had a history of drug use; and (5) have other known diseases or comorbidities that could affect the cortisol level. Finally, we recruited 200 PLWH as the study group and 20 people who were HIV-negative but their spouses were HIV-positive as the control group.

### Data collection procedure

Hair samples were collected from each participant by research staff from Department of Infectious Disease between November 2017 and April 2018. Guided by a standardized protocol as described previously^30^, two 1-cm hair samples (20–30 strands of hair) were cut as close to the scalp as possible from the vertex posterior region. The hair strands were cut with iron scissors which had been sanitized. The hair thatch, which was divided into two parts, was completely enclosed by a piece of foil with a small label indicating the study ID.

After the hair sample collection, all the participants must complete the survey to collect their sociodemographic characteristics and psychosocial outcomes (e.g. quality of life, anxiety, perceived stress and mental resilience). The information about hair care (e.g. frequency of hair washing, using hair dryer/curling iron or hair straightener or not, using hair styling products or not) was collected as well. Each interview lasted for 30 min and was performed through a one-on-one format. Interviewers were internal healthcare nurses who had received intensive training on research ethics and data collection with PLWH before the study.

### Cortisol assay

The hair cortisol was analyzed in Southeast University hair analytical laboratory using a developed and validated liquid chromatography tandem mass spectrometry (LC-MS/MS) method^31^.

Hair samples were washed twice with 3 mL methanol for 2 min and then dried at a room with temperature of 25℃ for at least 12 h. A sample of 20 mg hair was then incubated in 1 mL methanol with 1 ng cortisol-d4 as internal standard (IS) for 24 h at 25 °C. After being centrifuged at 12,000 rpm for 5 min, 800 μL of the clear supernatant was transferred into a new 2 mL tube and evaporated under nitrogen at 50 °C. The dry residue was re-suspended using 50 μL deionized water and 950 μL methanol for solid-phase extraction. The final eluate was evaporated to dryness, and re-suspended in 50 μL mobile phase for the next analysis.

The analysis was performed by liquid chromatography (1200 series, Agilent, Germany) and a mass spectrometry (3200 QTRAP, ABI, USA), equipped with an atmospheric pressure chemical ionization (APCI) source operating in the multiple reaction monitoring and positive mode. Nitrogen (99.999%) was selected as the nebulizing gas. The optimum ionization parameters followed the protocols that were reported in the previous studies^31,32^.

The current LC-APCI-MS/MS method showed the limits of detection (LOD) and quantification (LOQ) between 0.25 and 0.50 pg/mg for hair cortisol where LOD and LOQ were defined as the concentrations with a signal-to-noise ratio (S/N) between 3 and 10. Linearity was achieved at 0.5–250 pg/mg (r2 = 0.9983) of hair cortisol. Intra-day and inter-day precisions and recovery (n = 5) were evaluated at 0.5, 5 and 200 pg/mg. Intra- day and inter-day coefficients of variation were less than 10% at the three concentrations and recovery was more than 98%. The method validation of cortisol assay is based on the Bioanalytical Method Validation Guidance for Industry announced by Food and Drug Administration from the USA^33^.

## Measure

### Socio-demographic characteristics

Participants provided information about their socio-demographic characteristics including age, gender, ethnicity, marital status, education level, employment status, monthly household income, tobacco use, alcohol use, and hair care (e.g. using hairdryer/curling iron or hair straightener or not, using hair styling products or not). Participant also reported their estimated weight and height. We calculated Body Mass Index (BMI) based on weight and height for each participant using an established formula BMI = weight (kg)/[height(m)]^2^.

### Psychological measures

#### Quality of life

We employed Simplified Chinese Version of MOS-HIV Scale to evaluate the quality of live among all the participants. The MOS-HIV is a 35-item scale capturing 11 dimensions of health-related quality of life including physical functioning, role functioning, pain, social functioning, emotional well-being, energy/fatigue, cognitive functioning, general health, health distress, overall quality of life, and health transition. The summed score was calculated the quality of life, with higher score representing better functioning and well-being (Cronbach’s α = 0.938).

#### Anxiety

We employed the Zung Self-rating Anxiety Scale (SAS) to assess the levels of anxiety among participants. The SAS is a 20-item scale that assesses symptoms of anxiety with a 4-point response option. The sample items are “how often do you feel scared for no reason” and “how often do you feel all right”. SAS has been widely used in various Chinese populations and convincing internal reliability in the current study sample (Cronbach’s α = 0.93)^34^.

#### Perceived stress

Participants’ subjective experience of chronic stress was measured using the 14-item Perceived Stress Scale (PSS)^35^, which has been validated in a wide variety of populations^36^. The PSS is a global measure of subjective stress that assesses the extent to which respondents perceive their lives as being unpredictable, uncontrollable, or overwhelming in the last month. Each item is rated on a 5-point scale ranging from “almost never” to “almost always” with higher scores indicating higher levels of perceived stress (Cronbach’s α = 0.78).

#### Psychological resilience

The 10-item Connor-Davidson Resilience Scale (CD-RISC) was employed to evaluate the psychological resilience in this study. It assesses an individual’s ability to adapt to the adversities in the past month. The response option is a 5-point Likert scale ranging from 0 (not true at all) to 4 (true nearly all the time). The summed score was calculated to reflect the resilience capacity, which higher score indicating higher resilience (Cronbach’s α = 0.856).

### Propensity score matching (PSM)

The R version 3.6.3 software program (R Foundation for Statistical Computing, Vienna, Austria, available at https://www.r-project.org/) was used to perform PSM. To adjust for the potential confounders between study and control groups, we applied PSM which used the nearest-neighbor matching algorithm with a 1:3 matching scheme for matching via R package “Matching”. The propensity score was estimated using the 13 potential confounders (e.g. age, gender, BMI, ethnicity, marital status, education level, employment status, monthly household income, tobacco use, alcohol use, frequency of hair washing, using Hair dryer/curling iron or hair straightener and hair styling products). The 1:3 PSM matched 20 participants in the control group and 60 PLWH in the study group. Lastly, we examined the standardized differences among the 13 confounders before and after PSM using the R package “Tableone”.

### Statistical analysis

Descriptive statistics were reported on the variables of interest. Categorical variables were described using frequencies and percentages, whereas the continuous variables were described using median and interquartile range (IQR). Differences in quantitative results were analyzed using the Pearson’s Chi-squared or Fisher’s exact tests, while those in semi-quantitative results were analyzed using the Mann–Whitney U test.

We examined the Spearman correlations between hair cortisol and psychological measures. To assess the differences of psychological measures between different levels of hair cortisol, the hair cortisol level was categorized into to two sub-groups (“low group”: ≤ 29.56, “high group”: > 29.56)using the median values of cortisol. Mann–Whitney U test was employed to examine the associations between psychological measures and hair cortisol level.

All statistical analyses were conducted using the Statistical Pack for the Social Science (SPSS) version 23.0 software program (IBM Corp, Armonk, NY, USA). P values are two-tailed, and values less than 0.05 were considered statistically significant.

## References

[1] Ortblad KF (2013). The burden of HIV: insights from the Global Burden of Disease Study 2010. AIDS.

[2] https://apps.who.int/gho/data/view.main.22100WHO?lang=en

[3] McIntosh RC (2012). Stress and coping in women living withHIV: a meta-analytic review. AIDS Behav..

[4] Napravnik S, Royce R, Walter E, Lim W (2000). HIV-1 infected women and prenatal care utilization: barriers and facilitators. AIDS Patient Care STDs.

[5] Hellhammer DH, Wust S, Kudielka BM (2009). Salivary cortisol as a biomarker in stress research. Psychoneuroendocrinology.

[6] Miller GE, Chen E, Zhou ES (2007). If it goes up, must it come down? Chronic stress and the hypothalamic-pituitary-adrenocortical axis in humans. Psychol. Bull..

[7] Liu AY (2014). Strong relationship between oral dose and tenofovir hair levels in a randomized trial: hair as a potential adherence measure for pre-exposureprophylaxis (PrEP). PLoS ONE.

[8] Russell E (2015). Toward standardization of hair cortisol measurement: results of the first international interlaboratory round robin. Ther. Drug Monit..

[9] Chao H, Cui ZY, Wang Y, Li L, Huang HH (2017). Analysis of mental health status among people with HIV/AIDS. Chin. J. Hum. Sex..

[10] Sinicco A (1993). Cytokine network and acute primary HIV-1 infection. AIDS.

[11] Tao W, Feng XS, Wu YC (2004). A method to estimate the hazard ratio in non-randomized medical researches. Chi. J. Heal. Sta..

[12] Langerak T (2015). The relation between long-term cortisol levels and the metabolic syndrome in HIV-infected patients. Clin. Endocrinol..

[13] Gong SL (2003). Depression.

[14] Wan YJ, Dong HY, Zhan Y, Zhang RF, Lu LX (2007). The mental problems and needs in patients under AIDS/HIV discrimination. Chin. Rem. Clin..

[15] Chen QL (2004). Effect of mental health and psychosocial factors in individual with HIV/AIDS. Chin. Ment. Health.

[16] Antoni MH (2000). Cognitive-behavioral stress management reduces distress and 24-hour urinary free cortisol output among symptomatic HIV-infected gay men. Ann. Behav. Med..

[17] Villette, J.M. *et al*. Circadian variations in plasma levels of hypophyseal, adrenocortical and testicular hormones in men infected with human immunodeficiency virus. *J Clin EndocrinolMetab* **70**, 572–577 (1990). 10.1210/jcem-70-3-572 2155251

[18] Qiao S (2017). Hair measurements of cortisol, DHEA, and DHEA to cortisol ratio as biomarkers of chronic stress among people living with HIV in China: known-group validation. PLoS ONE.

[19] Odeniyi IA, Fasanmade OA, Ajala MO, Ohwovoriole AE (2013). Adrenocortical function in Nigerians with human immunodeficiency virus infection. Ghana Med. J..

[20] Zaid D, Greenman Y (2019). Human immunodeficiency virus infection and the endocrine system. Endocrinol. Meta.

[21] Dettenborn L (2012). Introducing a novel method to assess cumulative steroid concentrations: increased hair cortisol concentrations over 6 months in medicated patients with depression. Stress.

[22] Karlen J, Ludvigsson J, Frostell A, Theodorsson E, Faresjo T (2011). Cortisol in hair measured in young adults—a biomarker of major life stressors?. BMC Clin. Pathol..

[23] Steudte S (2011). Increased cortisol concentrations in hair of severely traumatized Ugandan individuals with PTSD. Psychoneuroendocrinology.

[24] Staufenbiel SM, Penninx BW, Spijker AT, Elzinga BM, van Rossum EF (2013). Hair cortisol, stress exposure, and mental health in humans: a systematic review. Psychoneuroendocrinology.

[25] Yekta D (2010). Relationship between hair cortisol concentrations and depressive symptoms in patients with coronary artery disease. Neuropsychiatr. Dis. Treat..

[26] Manenschijn L (2012). Long-term cortisol in bipolar disorder: associations with age of onset and psychiatric co-morbidity. Psychoneuroendocrinology.

[27] Steudte S (2011). Decreased hair cortisol concentrations in generalised anxiety disorder. Psychiatry Res..

[28] Tobias S, Frank H, Phil E, Angela C (2009). Use of a single case study design to examine state variation in the cortisol awakening response: relationship with time of awakening. Psychoneuroendocrinology.

[29] Akoku DA, Tihnje MA, Tarh EO, Tarkang EE, Mbu RE (2018). Predictors of willingness to accept pre-marital HIV testing and intention to sero-sort marital partners; risks and consequences: findings from a population-based study in Cameroon. PLoS ONE.

[30] Sauve B, Koren G, Walsh G, Tokmakejian S, Van Uum SH (2007). Measurement of cortisol in human hair as a biomarker of systemic exposure. Clin. Invest. Med..

[31] Chen Z (2019). Determination, intercorrelation and intraindividual stability of five steroids in hair, saliva and urine among Chinese college students. Steroids.

[32] Gao W (2013). Quantitative analysis of steroid hormones in human hair using a column-switching LC-APCI-MS/MS assay. J. Chromatogr. B Analyt. Technol. Biomed. Life Sci..

[33] https://www.fda.gov/regulatory-information/search-fda-guidance-documents/bioanalytical-method-validation-guidance-industry.

[34] Sun W, Wu M, Qu P, Lu C, Wang L (2014). Psychological well-being of people living with HIV/AIDS under the new epidemic characteristics in China and the risk factors: a population-based study. Int. J. Infect. Dis..

[35] Cohen S, Kamarck T, Mermelstein R (1983). A global measure of perceived stress. J. Health Soc. Behav..

[36] Lee EH (2012). Review of the psychometric evidence of the perceived stress scale. Asian Nurs. Res..

